# Brucella abortus Infection of Placental Trophoblasts Triggers Endoplasmic Reticulum Stress-Mediated Cell Death and Fetal Loss via Type IV Secretion System-Dependent Activation of CHOP

**DOI:** 10.1128/mBio.01538-19

**Published:** 2019-07-23

**Authors:** Mariana X. Byndloss, April Y. Tsai, Gregory T. Walker, Cheryl N. Miller, Briana M. Young, Bevin C. English, Núbia Seyffert, Tobias Kerrinnes, Maarten F. de Jong, Vidya L. Atluri, Maria G. Winter, Jean Celli, Renée M. Tsolis

**Affiliations:** a Department of Medical Microbiology and Immunology, School of Medicine, University of California at Davis, Davis, California, USA; University of Pittsburgh School of Medicine; University of Michigan Medical School; East Carolina University School of Medicine

**Keywords:** *Brucella*, type IV secretion, effector functions, endoplasmic reticulum, placenta, trophoblast

## Abstract

Subversion of endoplasmic reticulum (ER) function is a feature shared by multiple intracellular bacteria and viruses, and in many cases this disruption of cellular function activates pathways of the unfolded protein response (UPR). In the case of infection with Brucella abortus, the etiologic agent of brucellosis, the unfolded protein response in the infected placenta contributes to placentitis and abortion, leading to pathogen transmission. Here we show that B. abortus infection of pregnant mice led to death of infected placental trophoblasts in a manner that depended on the VirB type IV secretion system (T4SS) and its effector VceC. The trophoblast death program required the ER stress-induced transcription factor CHOP. While NOD1/NOD2 expression in macrophages contributed to ER stress-induced inflammation, these receptors did not play a role in trophoblast death. Both placentitis and abortion were independent of apoptosis-associated Speck-like protein containing a caspase activation and recruitment domain (ASC). These studies show that B. abortus uses its T4SS to induce cell-type-specific responses to ER stress in trophoblasts that trigger placental inflammation and abortion. Our results suggest further that in B. abortus the T4SS and its effectors are under selection as bacterial transmission factors.

## Abstract (precis)

Brucella abortus infects the placenta of pregnant cows, where it replicates to high levels and triggers abortion of the calf. The aborted material is highly infectious and transmits infection to both cows and humans, but very little is known about how B. abortus causes abortion. By studying this infection in pregnant mice, we discovered that B. abortus kills trophoblasts, which are important cells for maintaining pregnancy. This killing required an injected bacterial protein (VceC) that triggered an endoplasmic reticulum (ER) stress response in the trophoblast. By inhibiting ER stress or infecting mice that lack CHOP, a protein induced by ER stress, we could prevent death of trophoblasts, reduce inflammation, and increase the viability of the pups. Our results suggest that B. abortus injects VceC into placental trophoblasts to promote its transmission by abortion.

## INTRODUCTION

The placenta is an important site of infections that can result in spontaneous abortion, perinatal mortality of the infant, or vertical transmission to the developing fetus (1). Colonization of the placenta is central to the pathogenesis of viral diseases caused by Zika virus and cytomegalovirus, protozoan infection caused by Toxoplasma gondii, and bacterial infections such as Q fever, listeriosis, and brucellosis (1, 2). A shared feature of several of these pathogens is their ability to replicate within host cells, specifically within fetally derived trophoblasts, which depending on the host species are termed extravillous trophoblasts in humans, trophoblast giant cells in mice, or intercotyledonary trophoblasts in ruminants (3).

Brucella abortus infects the placenta of cows, where it replicates within intercotyledonary trophoblasts, triggering trophoblast necrosis and severe inflammatory pathology that is thought to cause abortion by disruption of the fetal-maternal interface (4, 5). In some cases, less severe placental pathology results in birth of weak calves and perinatal mortality or in vertical transmission of the infection to viable offspring. Zoonotic infection with *Brucella* spp. in pregnant women, while not extensively studied, has been reported in association with adverse pregnancy outcomes, including increased risk for miscarriage, preterm delivery, and vertical transmission to the fetus (6, 7).

The mechanisms by which B. abortus triggers inflammation in the placenta are poorly understood. Histologic evidence of trophoblast necrosis in infected ruminant placentas is at odds with the behavior of B. abortus in the mononuclear phagocyte system, where it elicits low-level granulomatous inflammation (8). A pregnant mouse model, in which B. abortus localizes within trophoblast giant cells, showed that inflammation is important for fetal loss, since neutralization of either gamma interferon or RANTES (regulated upon activation, normal T-cell expressed and secreted) improved fetal survival in pregnant mice infected with B. abortus (9, 10). It was concluded from these studies that induction of a systemic Th1 response by B. abortus during the early stage of pregnancy was responsible for fetal loss. Further, mutants attenuated for persistent infection in mice such as the vaccine strain S19 and the *virB4* mutant, deficient for the VirB type IV secretion system (T4SS), can replicate in the placenta without causing abortion (9, 10). However, it is unclear from these studies which bacterial factors mediate interactions with placental cells that result in abortion.

Recent work from our group and others showed that intracellular infection of macrophages with B. abortus or B. melitensis leads to induction of the IRE1α pathway of the cellular unfolded protein response (11–13). One of the B. abortus factors that activate the IRE1α pathway during macrophage infection is T4SS effector VceC (for VirB-coregulated effector C) (11). During macrophage infection by B. abortus, the innate immune receptors NOD1 and NOD2 sense IRE1α activation to initiate cellular production of proinflammatory cytokines (14). In pregnant mice both the VceC-induced unfolded protein response and its downstream NOD1/NOD2 signaling contribute to abortion, since deficiency of NOD1 and NOD2 or treatment with an inhibitor of endoplasmic reticulum (ER) stress increased fetal survival (14). However, NOD1/NOD2 knockout mice were not completely resistant to B. abortus-induced abortion, which prompted us to identify additional cellular pathways involved in triggering placental and fetal pathology.

## RESULTS

### Death of trophoblast giant cells during B. abortus infection results from endoplasmic reticulum stress.

**FIG 1:**
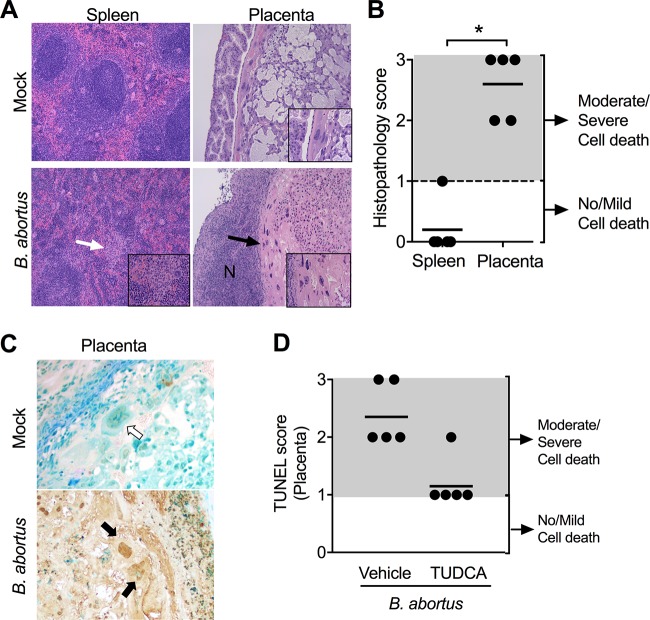
B. abortus infection induces cell death in the placenta. (A) Representative images of spleen and placenta from pregnant mice infected with wild-type B. abortus for 13 days. The white arrow and left bottom inset show microgranulomas, the black arrow and right bottom inset show trophoblast death, and “N” shows areas of neutrophilic infiltrate (20×). (B) Cell death assessed by blinded histopathology scoring in spleen and placenta from mice in panel A. Values represent individual mice (black circles) and means (black line). *, *P* < 0.05 using Mann-Whitney statistical analysis. (C) Representative images of TUNEL-stained placenta tissue from panel B. (D) Pathology scoring of TUNEL staining in placentas of B. abortus-infected mice treated with a vehicle or the ER stress inhibitor TUDCA.

B. abortus infects placental trophoblasts in cows and in experimentally inoculated goats, and histologic evaluation of these tissues reveals evidence of cell death. To determine whether this feature of ruminant infection can be modeled in the mouse, we analyzed histologic evidence of cell death in spleens and placentas of pregnant mice that were inoculated intraperitoneally (i.p.) with B. abortus at day 5 of gestation (Fig. 1A and B). While no histologic evidence of cell death was observed in splenic tissue, moderate to severe cell death was observed in the placenta, as evidenced by pyknotic nuclei and cytoplasmic acidification in trophoblasts, suggesting that trophoblast death was also occurring during placental infection. Both trophoblast giant cells and infiltrating neutrophils in infected placentas stained positive for terminal deoxynucleotidyl transferase (TdT) dUTP nick end labeling (TUNEL), which detects nuclear DNA fragmentation that occurs during apoptotic cell death (Fig. 1C).

Since our previous work implicated ER stress in both splenic inflammation and abortion in B. abortus-infected mice (11, 14), we asked whether trophoblast death resulted from ER stress. Pregnant mice were treated with a vehicle or with tauroursodeoxycholic acid (TUDCA), which alleviates ER stress by promoting protein folding. Blinded scoring of TUNEL staining in cell nuclei of placental tissue revealed moderate to severe trophoblast death in placentas from B. abortus-infected mice treated with the vehicle, whereas treatment with TUDCA strongly reduced trophoblast death, as evidenced by a reduction in TUNEL staining (Fig. 1D).

### The T4SS effector VceC contributes to trophoblast killing and fetal loss in a pregnant mouse model of B. abortus infection.

**FIG 2:**
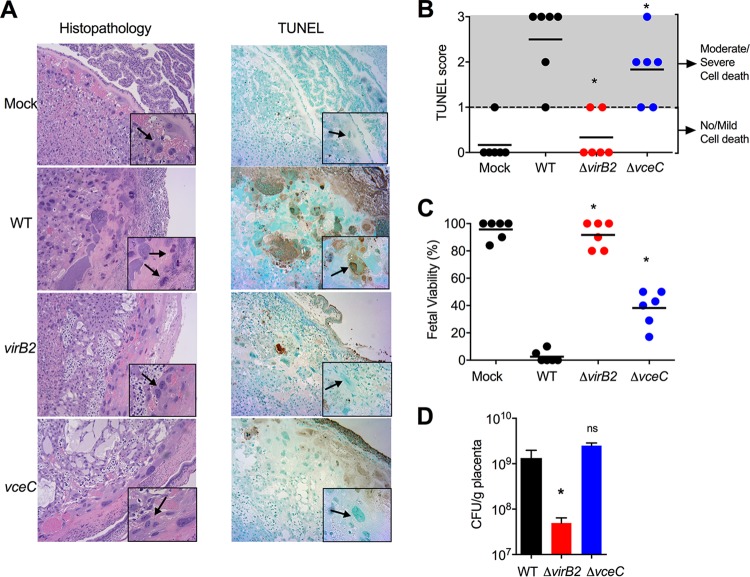
Death of trophoblast giant cells is dependent on the T4SS and its effector VceC. (A) Representative images of hematoxylin and eosin-stained (left) and TUNEL-stained (right) placental tissue from mock-infected or B. abortus-infected pregnant mice. The black arrow and inset show trophoblasts (20×). (B) Trophoblast death measured by TUNEL assay in placenta from pregnant mice infected with wild-type (WT) B. abortus or isogenic *virB2* and *vceC* mutants for 13 days (*n* = 6). Values represent individual mice (black circles) and means (black lines). *, *P* < 0.05 using Mann-Whitney statistical analysis. (C) Fetal viability in pregnant mice infected with wild-type B. abortus or isogenic *virB2* and *vceC* mutants for 13 days (*n* = 6). Values represent individual mice (black circles) and means (black lines). *, *P* < 0.05 using one-way ANOVA. (D) Placental colonization with wild-type B. abortus or its isogenic *virB2* and *vceC* mutants in pregnant mice infected for 13 days. Values represent means ± SEM. *, *P* < 0.05 using one-way ANOVA. ns, nonsignificant.

Our previous work implicated the T4SS effector VceC, which localizes to the endoplasmic reticulum and interacts with the ER chaperone BiP, in placentitis and abortion caused by B. abortus (14). To determine whether the T4SS and VceC played a role in trophoblast death, we compared cell death *in situ* in placentas from mice infected with either wild-type B. abortus, a *virB2* mutant defective for assembly of the T4SS apparatus, or a *vceC* mutant (Fig. 2A and B). As shown above (Fig. 1C), placentas from mice infected with wild-type B. abortus exhibited histologic evidence of moderate to severe cell death (Fig. 2A). In contrast, an absence of placental cell death was noted in mice infected with the *virB2* mutant. Placentas from mice infected with the *vceC* mutant exhibited an intermediate phenotype, suggesting that VceC contributes to placental cell death during B. abortus infection (Fig. 2A). To gain a second line of evidence for death of trophoblasts, we performed TUNEL staining to detect nuclear DNA fragmentation. Blinded scoring of TUNEL staining in cell nuclei provided evidence for moderate to severe trophoblast death in placentas from mice infected with wild-type B. abortus. However, trophoblast death was significantly reduced in mice infected with the *vceC* mutant and absent in mice inoculated with the *virB2* mutant (Fig. 2A and B). The TUNEL score corresponded with fetal viability in the mice (represented as the percentage of viable pups in the litter of each dam in the experiment), with *virB2* mutant-infected mice showing no reduction in fetal viability compared to mock-infected mice and the *vceC* mutant-infected mice exhibiting an intermediate level of fetal death (Fig. 2C). The inability of the *virB2* mutant to cause fetal death correlated with reduced placental colonization, whereas deletion of *vceC* did not reduce fitness of B. abortus in the placental infection niche (Fig. 2D). These results suggested that the T4SS, perhaps via additional effectors, is involved in placental colonization, whereas VceC induces placentitis and cell death independently of an effect on colonization.

### Death of trophoblast giant cells during B. abortus infection does not require NOD1 and NOD2.

**FIG 3:**
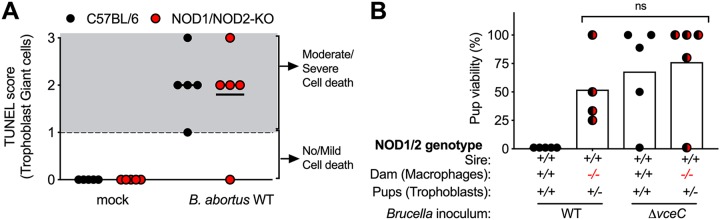
Role of NOD1 and NOD2 in death of trophoblast giant cells. (A) Role of NOD1/NOD2 in trophoblast death. *Nod1*^+/+^ *Nod2*^+/+^ or *Nod1*^−/−^ *Nod2*^−/−^ dams were bred to sires of the same genotype and inoculated with B. abortus. Trophoblast death in the placentas at 13d postinfection was assessed by TUNEL staining. (B) Contribution of NOD1/NOD2 in maternally derived immune cells to B. abortus-induced abortion. *Nod1*^+/+^ *Nod2*^+/+^ or *Nod1*^−/−^ *Nod2*^−/−^ dams (*n* = 4–5) were bred to *Nod1*^+/+^ *Nod2*^+/+^ sires to generate fetuses carrying immune cells with the maternal *Nod1 Nod2* genotype. Each dot represents the percent viability of pups in one litter and the bar indicates the geometric mean of the group.

Our previous results showed a role for NOD1 and NOD2 in abortion caused by B. abortus in mice, as mice deficient for both NOD1 and NOD2 had reduced inflammation and increased viability of pups after infection (14). This work showed a role for NOD1 and NOD2 in triggering inflammation in response to activation of the IRE1α pathway of the unfolded protein response (UPR). However, it was not clear from this study whether NOD1 and NOD2 had a role in death of trophoblast giant cells. To answer this question, we performed TUNEL assays on sections of placenta from control mice or NOD1/NOD2-deficient mice infected with B. abortus 2308. Blinded histopathology scoring revealed no difference in trophoblast death between the two groups (Fig. 3A), with similar levels of colonization (14). This result suggested that NOD1 or NOD2 is important for the phagocyte response to B. abortus infection, rather than contributing to trophoblast death. To examine this differently, we crossed *Nod1^−/−^ Nod2^−/−^* dams with wild-type sires to generate pregnant mice in which placental macrophages, which are derived from maternal tissue, are deficient for NOD1 and NOD2 and trophoblasts, which are derived from fetal tissue, have a *Nod1^+/−^ Nod2^−/−^* genotype and express NOD1 and NOD2 (Fig. 3B). Lack of NOD1 and NOD2 expression in macrophages was sufficient to recapitulate the increase in pup viability that we previously showed for B. abortus-infected *Nod1^−/−^ Nod2^−/−^* mice (14), suggesting a cell-type-specific role for NOD1 and NOD2 in placentitis and abortion. Interestingly, ASC, which has also been implicated in B. abortus-induced inflammatory responses at other sites (15, 16), was not required for placentitis or abortion (see Fig. S1 in the supplemental material).

10.1128/mBio.01538-19.1 FIG S1 Absence of effect of ASC inflammasomes on B. abortus-induced placentitis and abortion. Download FIG S1, PDF file, 0.2 MB. Copyright © 2019 Byndloss et al. 2019 Byndloss et al. This content is distributed under the terms of the Creative Commons Attribution 4.0 International license.

### Reduced cytotoxicity of the *vceC* mutant for trophoblasts *in vivo* does not result from defective intracellular trafficking.

**FIG 4:**
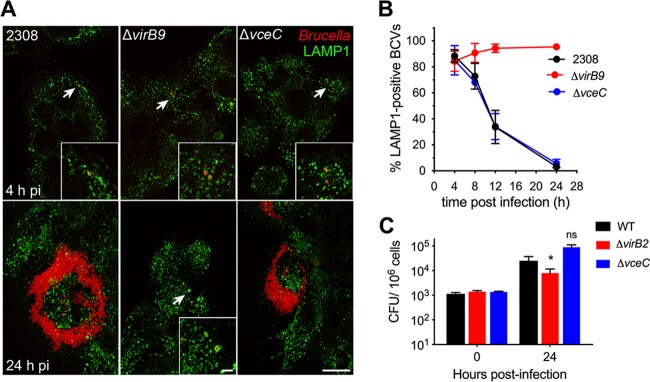
The T4SS effector VceC is not required for intracellular replication in BeWo trophoblast-like cells. (A) Fluorescence images of BeWo cells infected with B. abortus 2308 and isogenic Δ*virB9* and Δ*vceC* mutants (red) showing colocalization with LAMP-1 (green) at 4 h and 24 h postinfection. Scale bar in lower right panel represents 10 μm, and scale bar in inset represents 2 μm. Images are representative of three independent replicates. (B) Quantification of LAMP-1-positive BCVs in BeWo cells infected with B. abortus 2308 or Δ*virB9* and Δ*vceC* mutants at 4, 8, 12, and 24 h after B. abortus infection. Data are compiled from three independent experiments per strain and time point. (C) CFU of wild-type B. abortus, the Δ*virB* mutant, and the Δ*vceC* mutant in BeWo cells infected for 0 h and 24 h (*n* = 4). Values represent means ± SEM. *, *P* < 0.05 using one-way ANOVA.

VceC does not contribute to evasion of lysosomal degradation by B. abortus in macrophages (11); however, we wanted to determine whether the noncytotoxic phenotype might result from altered trafficking in trophoblasts. The BeWo choriocarcinoma line has been shown to model trafficking of B. abortus in placental trophoblasts (17), in which B. abortus replicates within the endoplasmic reticulum (18–20). Therefore, we utilized these cells to determine whether VceC contributes to exclusion of the phagolysosomal marker LAMP-1 from the *Brucella*-containing vacuole, a T4SS-dependent process that is required for B. abortus to replicate in its endoplasmic reticulum-associated niche (21). In contrast with a T4SS-defective *virB9* mutant, which remained LAMP-1 associated, the *vceC* mutant was able to exclude LAMP-1 to the same extent as wild-type B. abortus (Fig. 4A and B) and replicated to wild-type levels intracellularly. B. abortus did not reproducibly cause death of BeWo cells; therefore, these cells were not an appropriate model to study the cytotoxic phenotype of VceC observed in trophoblasts *in vivo*.

### Induction of CHOP contributes to VceC-mediated trophoblast death in the placenta.

**FIG 5:**
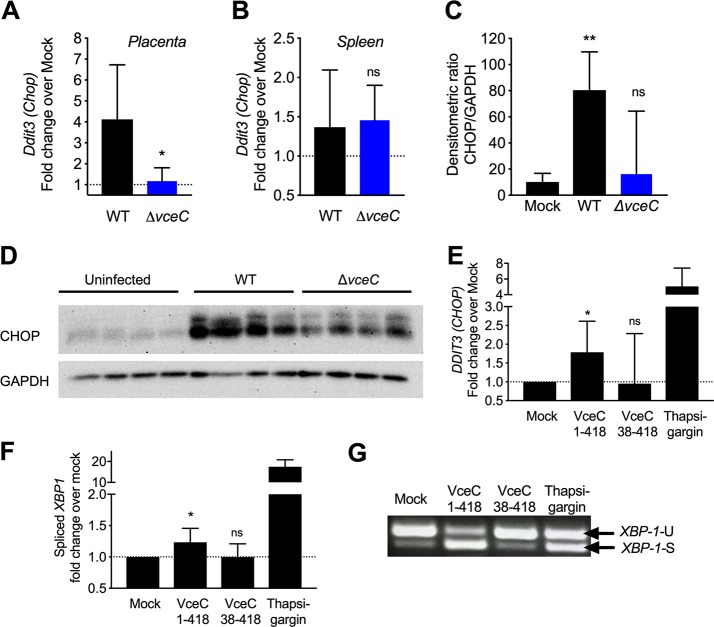
VceC induces expression of CHOP in the placenta. (A and B) RT-PCR analysis of transcripts for CHOP (*Ddit3*) in placentas (A) and spleens (B) of pregnant mice infected with wild-type B. abortus or the *vceC* mutant for 13 days (*n* = 7). Values represent means ± SEM. *, *P* < 0.05 using unpaired *t* test. (C and D) Detection of CHOP by Western blotting in placentas of uninfected mice or mice infected with wild-type B. abortus or the *vceC* mutant for 13 days (*n* = 4). (C) Densitometric quantification of CHOP signal relative to glyceraldehyde-3-phosphate dehydrogenase (GAPDH). Significance of differences was determined by a Kruskal-Wallis test with Dunn’s *post hoc* test. *, *P* < 0.05. (D) Western blots used for quantification in panel C. (E to G) Induction of ER stress responses in HEK293 cells after ectopic expression of VceC_1-418_ or VceC_38-418_ or after thapsigargin treatment. (E) Expression of *DDIT3* measured by qRT-PCR. Bars represent geometric means ± SEM. (F) Abundance of spliced *XBP1* transcript measured by qRT-PCR. Bars represent geometric means ± SEM. (G) Agarose gel showing spliced (S) and unspliced (U) *XBP1* transcripts.

Our previous work showed that B. abortus infection of murine bone marrow-derived macrophages led to activation of the IRE1α pathway, but not to death of infected cells (11). However, a second ER stress-induced pathway involving protein kinase R-like endoplasmic reticulum kinase (PERK) induces expression of *Ddit3*, encoding CCAAT/enhancer-binding protein (C/EBP) homologous protein (CHOP). Since CHOP is a mediator of ER stress-induced cell death (22), we investigated whether it mediated trophoblast death during placental infection (Fig. 5). In pregnant mice infected with wild-type B. abortus, an approximately 4-fold upregulation of *Ddit3* transcription was observed in the placenta, but this was absent in mice infected with the *vceC* mutant (Fig. 5A). This response was specific to the placenta, as no VceC-dependent induction of *Ddit3* expression was observed in the spleen (Fig. 5B). Increased abundance of CHOP was also observed in the placentas of B. abortus-infected pregnant mice, and this was dependent on expression of *vceC* (Fig. 5C and D). Together, these results showed that during placental infection, VceC elicits induction of CHOP. To determine whether VceC was sufficient to increase CHOP production, we utilized an ectopic expression model in HEK293 cells. Our previous work showed that the N terminus of VceC is needed to target it to the ER membrane and that deletion of this domain changes the localization of VceC from the ER to the cytosol (11). We used this feature to compare *DDIT3* transcription in cells ectopically expressing full-length VceC (VceC_1-418_) or N-terminally truncated VceC (VceC_38-418_). Significant induction of *DDIT3* transcription was observed only in cells expressing ER-targeted VceC (Fig. 5E). Similarly, ectopic expression of VceC_1-418_ induced splicing of *XBP1* (Fig. 5F and G), indicating that VceC targeting to the ER is sufficient to drive activation of the UPR.

### CHOP contributes to abortion and death of placental trophoblasts during B. abortus infection.

**FIG 6:**
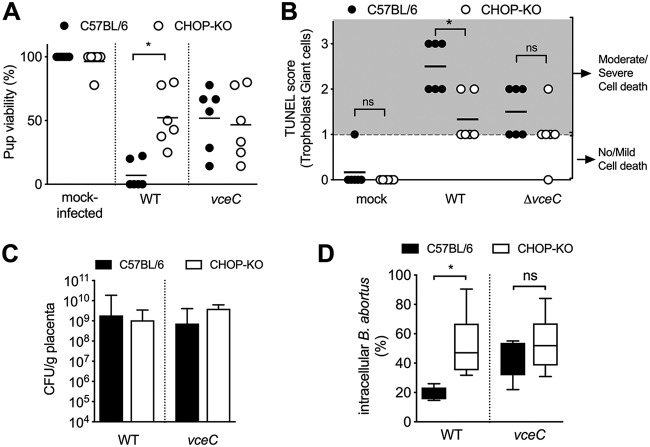
CHOP induction in the placenta contributes to fetal loss, trophoblast death, and extracellular release of B. abortus. (A) Viability of pups born to C57BL/6J or congenic CHOP knockout (CHOP-KO) dams infected for 13 days with B. abortus 2308 or isogenic *vceC* mutant (*n* = 4 for the mock-infected and *n* = 6 for the infected group). (B) Histologic scoring of TUNEL staining of trophoblasts from CHOP-KO mice. (C) B. abortus colonization of the placentas in mice from panel A. (D) Proportion of intracellular (gentamicin-resistant) versus extracellular (gentamicin-sensitive) B. abortus organisms after incubation of tissue *ex vivo* for 30 min in 50 mg/ml of gentamicin (*n* = 6). *, *P* < 0.05. Significance of differences was analyzed using a Mann-Whitney test.

Given the links between CHOP and ER stress-mediated cell death, we asked whether induction of CHOP in the placenta could lead to abortion. To test this idea, we inoculated *Chop*^−/−^ mice with either wild-type B. abortus or the *vceC* mutant. CHOP-deficient mice had greater fetal viability than congenic controls when inoculated with wild-type B. abortus. In contrast, no effect on fetal viability was observed after inoculation with the *vceC* mutant (Fig. 6A), which was consistent with the requirement of VceC for maximal CHOP activation (Fig. 5A and C). Further, placental tissues from infected CHOP-deficient mice contained fewer TUNEL-positive trophoblast giant cells (Fig. 6B), implicating CHOP in trophoblast death. Deficiency in CHOP did not affect the ability of B. abortus to replicate in the placenta (Fig. 6C). Remarkably, however, it altered the distribution of B. abortus between the intracellular and extracellular placental niches. *Ex vivo* treatment of placentas from infected mice with gentamicin, which acts preferentially on extracellular B. abortus, revealed that in C57BL/6 mice only a minority (20%) of B. abortus was intracellular (Fig. 6D). In contrast, in CHOP-deficient mice, the proportion of intracellular bacteria increased to approximately 50%. This difference was not observed in mice infected with the *vceC* mutant, in which a greater proportion of the bacteria were already in a gentamicin-protected niche (Fig. 6D). These results show that VceC-mediated ER stress in trophoblast giant cells of the placenta leads to induction of CHOP, which, in turn, triggers cell death and consequent release of bacteria to the extracellular space.

## DISCUSSION

Our results show that the B. abortus T4SS and its translocated effector VceC elicit ER stress in placental trophoblasts, resulting in cell death and induction of placental inflammation, two responses that promote fetal loss and transmission in cattle, the zoonotic reservoir host (5). These findings build on previous studies by others, including Anderson et al., who noted an early localization of B. abortus to placental trophoblasts in infected pregnant goats, with intracellular replication followed by trophoblast necrosis and necrotic placentitis (19), and Wang et al., who replicated this finding in immortalized goat trophoblasts (23). Several important pathological features of bovine infection, including inflammatory pathology, trophoblast death, and fetal death, are reproduced in the pregnant mouse (9, 14), suggesting that despite the different placentation types of mice and ruminants, the mouse can be used to study these features of placental B. abortus infection. The massive inflammatory and necrotic responses that are characteristic of placental B. abortus infection stand in stark contrast to pathology in the mononuclear phagocyte system, where B. abortus is localized within phagocytes and elicits a mild granulomatous inflammation (8). Notably, B. abortus, B. melitensis, and B. suis actually inhibit the death of infected macrophages (24, 25), which may serve to dampen inflammation and promote bacterial persistence in the mononuclear phagocyte system until the host becomes susceptible to placental colonization during pregnancy.

While B. abortus induces ER stress pathways in both macrophages and trophoblasts, a key difference in the cellular response to infection was the induction of CHOP in trophoblasts, which can be induced in response to ER stress via both IRE1α and PERK pathways (Fig. 6). CHOP is a transcription factor of the CCAAT/enhancer-binding protein (C/EBP) family that is induced in response to physiological changes in the cell, including ER stress, DNA damage, nutrient deprivation, hypoxia, and viral and fungal infection (26, 27). The downstream transcriptional responses mediated by CHOP can facilitate cell survival; however, severe or prolonged stress can overwhelm the capacity of this response to mitigate these insults and can activate cell death pathways (28). Upstream of CHOP, inhibition of IRE1α or alleviation of ER stress by TUDCA, a chemical chaperone, reduced cell death, implicating IRE1α in the trophoblast death response. Interestingly, CHOP deficiency in mice did not completely rescue fetal viability in B. abortus-infected pregnant mice, suggesting that other pathways are involved. One candidate for this pathway could be the IRE1α-XBP1-NLRP3-caspase 2 pathway, which was shown to mediate death of macrophages in response to infection with the B. abortus vaccine strain RB51 (29). However, it remains to be determined whether ER stress pathways are induced during infection of the bovine placenta and whether induction of these pathways contributes to trophoblast death and abortion in cattle.

A molecular mechanism by which VceC induces activation of CHOP during B. abortus infection is suggested by our previous work showing that ectopically expressed VceC localizes to the endoplasmic reticulum, where it interacts with the luminal chaperone BiP/GRP78 (11). During homeostasis, BiP interacts with the ER transducers of the cellular unfolded protein response, IRE1α, ATF6, and PERK, to maintain them in an inactive state (30). Accumulation of unfolded protein in the ER lumen titrates BiP away from the signal transducers, initiating the UPR. If VceC injected into B. abortus-infected trophoblasts by the T4SS behaves in the same manner as ectopically expressed VceC, it could modulate the activity of BiP, thereby inducing activation of the UPR signaling pathways. The ensuing death of trophoblasts appears to release B. abortus to an extracellular placental niche, where it can replicate and elicit inflammatory responses implicated in abortion. It will be of interest to determine whether additional B. abortus effectors that target the endoplasmic reticulum or induce ER stress also contribute to abortion and serve as transmission factors (13, 31). Taken together, these findings raise new questions on the mechanisms by which trophoblast death and release of bacteria elicit placental inflammation and fetal death.

This work has increased our understanding of how intracellular infection of placental trophoblasts leads to an adverse reproductive outcome. The ability of the placenta to restrict fetal infection is critical to successful reproduction, yet many pathogens are able to breach the placental barrier to infect fetal tissues (reviewed in reference 1). Fetally derived trophoblasts, in particular extravillous trophoblasts, are preferentially targeted by multiple pathogens in addition to B. abortus, including Listeria monocytogenes (32), Chlamydia pneumoniae (33), and Toxoplasma gondii (34), as well as viral pathogens such as Zika virus (35) and human cytomegalovirus (36). Collectively, these infections increase the risk for adverse pregnancy outcomes, including preeclampsia, abortion, preterm delivery, and perinatal infection. Considering the links between ER stress in trophoblasts and pathogenesis of preeclampsia (37), our findings of CHOP-mediated trophoblast death during B. abortus infection may have implications for understanding other placental pathologies as well.

## MATERIALS AND METHODS

### Bacterial strains, media, and culture conditions.

Bacterial strains used in this study were the virulent strain B. abortus 2308 and isogenic mutants carrying deletions in *virB2* (ADH3 [38]) and *vceC* (MDJ32 [39]). The *vceC* mutation was complemented using a plasmid-encoded copy of *vceC*, which was constructed by amplifying *vceC* and the 500-bp upstream promoter region from B. abortus (2308) genomic DNA. See Table S1 for primer sequences. The product was ligated into SalI-digested pBBR1MCS4 using the Gibson Assembly cloning kit (New England BioLabs [NEB]). The resulting construct was introduced into MDJ32 by electroporation. Expression of *vceC* in the complemented strains was not stably maintained; therefore, it was necessary to retransform B. abortus with plasmid-borne *vceC* prior to each experiment. For cellular infections, B. abortus was cultured on tryptic soy agar (TSA; Difco/Becton, Dickinson, Sparks, MD) or in tryptic soy broth at 37°C on a rotary shaker (at 200 rpm). Bacterial inocula for mouse infection were cultured on tryptic soy agar plus 5% blood for 3 days. All work with B. abortus cells was performed at biosafety level 3.

10.1128/mBio.01538-19.2 TABLE S1 Primers used in this study (strain construction and qPCR). Download Table S1, PDF file, 0.02 MB. Copyright © 2019 Byndloss et al. 2019 Byndloss et al. This content is distributed under the terms of the Creative Commons Attribution 4.0 International license.

### Ethics statement.

Experiments with mice were carried out in strict accordance with the recommendations in the *Guide for the Care and Use of Laboratory Animals* (40) and were approved by the Institutional Animal Care and Use Committee at the University of California at Davis under protocol number 17701.

### Animal experiments.

For the mouse placentitis model (14), C57BL/6J mice were used at an age of 8 to 10 weeks. ASC-deficient mice were provided by V. Dixit at Genentech (41). Mice were held in microisolator cages with sterile bedding and irradiated feed in a biosafety level 3 laboratory. Female C57BL/6J were mated with male C57BL/6J mice, and pregnancy was confirmed by the presence of a vaginal plug. At 5 days of gestation, groups of 5 to 7 pregnant mice were mock infected or infected intraperitoneally (i.p.) with 10^5^ CFU of B. abortus 2308 or its isogenic *virB2* or *vceC* mutant (day 0). At 13 days after infection (18 days of pregnancy), mice were euthanized by CO_2_ asphyxiation and the spleen and placenta were collected aseptically at necropsy. At day 13 postinfection (p.i.), pup viability was evaluated based on the presence of fetal movement and heartbeat and on fetal size and skin color (Table S2), and percentviability was calculated using the following formula: (number viable pups per litter/total number pups per litter) × 100. At necropsy, placenta samples were collected for bacteriology, gene expression analysis, and histopathological and TUNEL analyses. When indicated, mice were treated i.p. at days 10, 12, and 14 postinfection with a single dose of 250 mg/kg of body weight of TUDCA (Sigma-Aldrich, St. Louis, MO) or a vehicle control. Criteria for placental histopathology scoring are provided in Table S3.

10.1128/mBio.01538-19.3 TABLE S2 Criteria for evaluation of fetal viability. Download Table S2, PDF file, 0.01 MB. Copyright © 2019 Byndloss et al. 2019 Byndloss et al. This content is distributed under the terms of the Creative Commons Attribution 4.0 International license.

10.1128/mBio.01538-19.4 TABLE S3 Placental histopathology scoring criteria. Download Table S3, PDF file, 1.1 MB. Copyright © 2019 Byndloss et al. 2019 Byndloss et al. This content is distributed under the terms of the Creative Commons Attribution 4.0 International license.

For *ex vivo* gentamicin treatment, spleen and placentas from pregnant mice infected with B. abortus were collected in a 15-ml conical tube containing 1 ml of sterile phosphate-buffered saline (PBS), and the tissue was homogenized. The overall number of viable bacteria in the tissue (extracellular and intracellular B. abortus) was determined by performing serial 10-fold dilutions in sterile PBS and plating on TSA. In order to determine the overall number of intracellular viable bacteria in tissue, 100 μl of the initial tissue homogenate was transferred to 900 μl of sterile solution containing 50 mg/ml of gentamicin (Invitrogen, Grand Island, NY). The samples were incubated on ice for 30 min, followed by performing serial 10-fold dilutions in sterile PBS and plating on TSA. The overall number of viable extracellular bacteria in the tissue was calculated as follows: number of viable overall bacteria – number of viable intracellular bacteria (gentamicin treatment).

### qRT-PCR and data analysis.

Eukaryotic gene expression was determined by quantitative real-time PCR (qRT-PCR) as previously described (42). Briefly, eukaryotic RNA was isolated using TRI reagent (Molecular Research Center, Cincinnati, OH) according to the manufacturer’s instructions. A reverse transcriptase reaction was performed to prepare cDNA using TaqMan reverse transcription reagents (Applied Biosystems, Carlsbad, CA). A volume of 4 μl of cDNA was used as the template for each real-time PCR in a total reaction volume of 25 μl. Real-time PCR was performed using SYBR green (Applied Biosystems) and primers listed in Table S1. Data were analyzed using the comparative threshold cycle (*C_T_*) method (Applied Biosystems). Transcript levels of *Ddit3* (encoding CHOP), *Hspa5* (encoding BiP/GRP78), and *Xbp1* were normalized to mRNA levels of the housekeeping gene *Actb* in mouse samples.

### XBP1 splicing assay.

RNA from HEK293T cells transfected with VceC_1-418_, VceC_28-148_, or the vector control was isolated and reverse transcribed to cDNA as described above. Spliced *XBP1* was amplified from cDNA prepared from HEK293T cells and using hXBP1 primers (Table S1). Spliced and unspliced products were resolved on a 2.5% polyacrylamide gel in Tris-borate-EDTA.

### Western blotting.

Proteins were extracted from placentas using Tri reagent (Sigma-Aldrich) following the manufacturer’s protein extraction protocol, normalized by bicinchoninic acid (BCA), resolved by SDS-PAGE, and transferred to polyvinylidene difluoride (PVDF) membranes. CHOP was detected using mouse anti-CHOP antibody (CST 2895) horseradish peroxidase (HRP)-conjugated goat anti-mouse (Jackson ImmunoResearch) secondary antibody.

### Histopathology.

Formalin-fixed sections of spleen and placenta were stained with hematoxylin and eosin, and a veterinary pathologist performed a blinded evaluation using previously described criteria (14). Trophoblast death was determined as a cell presenting a highly basophilic pyknotic nucleus and acidophilic cytoplasm, and a score from 0 to 3 was given according to the intensity and distribution of dead cells in the tissue (0, no cell death; 1, mild focal cell death; 2, moderate, multifocal cell death; and 3, severe, multifocal to diffuse cell death). Representative images were obtained using a Zeiss Primo Star microscope with the brightness adjusted (Adobe Photoshop CS2).

### TUNEL assay.

Trophoblast death in formalin-fixed sections of placenta was determined by a terminal deoxynucleotidyl transferase (TdT) dUTP nick-end labeling (TUNEL) assay using the ApopTag peroxidase *in situ* apoptosis detection kit (Millipore, Billerica, MA) following the manufacturer’s protocol. A veterinary pathologist performed a blinded evaluation, and a cell death score from 0 to 3 was assigned according to the intensity and distribution of dead cells in the tissue as described above. Representative images were obtained using a Zeiss Primo Star microscope with the brightness adjusted (Adobe Photoshop CS2).

### Immunofluorescence microscopy.

BeWo cells seeded onto 12-mm glass coverslips were infected with DsRed*_m_*-expressing B. abortus strains and processed for immunofluorescence staining as follows. Coverslips were washed three times in 1× PBS and then fixed in 3% paraformaldehyde (EMD) in 1× PBS for 20 min at 37°C. Samples were then washed three times with 1× PBS, and free aldehydes were quenched in 50 mM ammonium chloride in PBS for 30 min at room temperature. Samples were blocked and permeabilized for 30 min in 0.1% (wt/vol) saponin, 10% (vol/vol) normal horse serum, and 1× PBS and then incubated for 1 h with mouse anti-human LAMP-1 H4A3 antibody (deposited to the Developmental Studies Hybridoma Bank by J. T. August and J. E. K. Hildreth) diluted in permeabilization buffer at room temperature. Samples were washed in 0.1% saponin-PBS and then 1× PBS and incubated for 30 min with Alexa Fluor 488-conjugated donkey anti-mouse IgG antibodies (1:500; Invitrogen, Life Technologies) at room temperature. Coverslips were washed in PBS, then rinsed in distilled H_2_O, and mounted on glass slides in Mowiol (Calbiochem). Samples were viewed with a Leica DM4000 epifluorescence upright microscope for quantitative analysis or a Leica SP8 confocal laser-scanning microscope for image acquisition. Representative confocal micrographs of 1,024 by 1,024 pixels were acquired and assembled using Adobe Photoshop CS6. Quantification of LAMP-1-positive vacuoles was performed as described previously (43).

### Statistical analysis.

Bacterial counts and fold changes of ratios (mRNA levels) and percentages (fetal viability) were transformed logarithmically prior to statistical analysis. An unpaired Student *t* test (between 2 groups) or one-way analysis of variance (ANOVA) followed by Tukey’s honestly significant difference (HSD) test (between >2 groups) was performed on the transformed data to determine whether differences between groups were statistically significant (*P* < 0.05). Significance of differences in histopathology and TUNEL scores was determined by a one-tailed nonparametric test (Mann-Whitney).
