# Supplementary material for: Brucella abortus Infection of Placental Trophoblasts Triggers Endoplasmic Reticulum Stress-Mediated Cell Death and Fetal Loss via Type IV Secretion System-Dependent Activation of CHOP
Source: mBio. 2019 Jul 23;10(4):e01538-19. doi: 10.1128/mBio.01538-19 (PMC6650558; doi:10.1128/mBio.01538-19)
Supplement: FIG S1 [file mBio.01538-19-sf001.pdf]

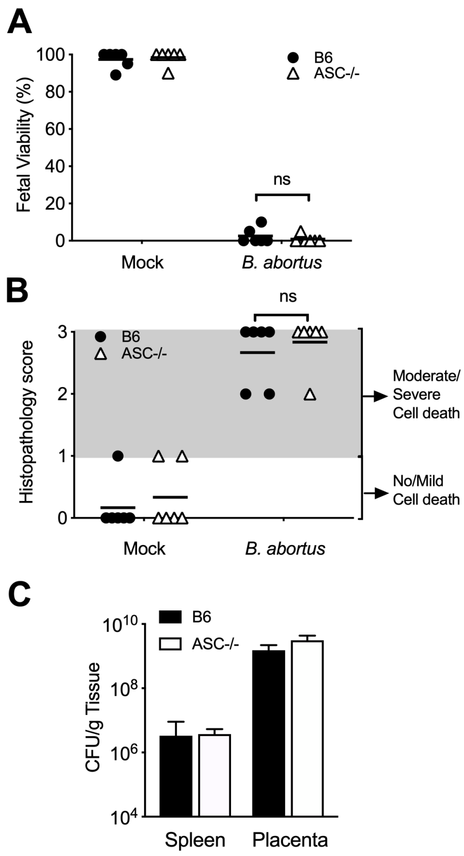

Figure S1. *Brucella abortus* induced trophoblast killing and fetal loss is independent of Caspase-1 activation. (**A**) Fetal viability from pregnant C57BL/6J WT (WT) or ASC-deficient (ASC-/-) mice infected with wild-type *B. abortus* for 13 days n=6. Values represent individual mice (black circles for WT, white triangles for ASC-/-) and mean (black dash). (**B**) Trophoblast cell death measured by histopathology scoring in placenta from pregnant C57BL/6J WT (WT) or ASC-deficient (ASC-/-) mice infected with wild-type *B. abortus* for 13 days n=6. Values represent individual mice (black circles for WT, white triangles for ASC-/-) and mean (black dash). *P<0.05 using Mann-Whitney statistical analysis. (**C**) Colonization of spleens and placentas of Asc-deficient mice after infection with *B. abortus* 2308.
