# Supplementary material for: Brucella abortus Infection of Placental Trophoblasts Triggers Endoplasmic Reticulum Stress-Mediated Cell Death and Fetal Loss via Type IV Secretion System-Dependent Activation of CHOP
Source: mBio. 2019 Jul 23;10(4):e01538-19. doi: 10.1128/mBio.01538-19 (PMC6650558; doi:10.1128/mBio.01538-19)
Supplement: TABLE S1 [file mBio.01538-19-st001.pdf]

Table S1: Sequences of oligonucleotide primers used in this study

Insert	Primer	Primer sequence (5'->3')
500 bp *vceC* promoter	F_500bp	CAAGCTTATCGATACCGTCGACTGTCGCGACATCAAGCCAAC
	R_500bp	TTCCTTCATGCGGATACCCTCTTACACTATAAAC
vceC	F_ vceC	GTATCCGCATGAAGGAATGGCTCAGC
	R_ vceC	GGCCCCCCCTCGAGGCTAATTGCGGGTTTCTCC
human *DDIT3*	hChop_F	GCACCTCCCAGAGCCCTCACTCTCC
	hChop_R	GTCTACTCCAAGCCTTCCCCCTGCG
human *XBP1*	hXbp1_F	AGCTCAGACTGCCAGAGATCG
	hXbp1_R	AATCCATGGGGAGATGTTCTA
mouse *Ddit3*	mChop_F	CTGGAAGCCTGGTATGAGGAT
	mChop_R	CAGGGTCAAGAGTAGTGAAGGT
