# Supplementary material for: Brucella abortus Infection of Placental Trophoblasts Triggers Endoplasmic Reticulum Stress-Mediated Cell Death and Fetal Loss via Type IV Secretion System-Dependent Activation of CHOP
Source: mBio. 2019 Jul 23;10(4):e01538-19. doi: 10.1128/mBio.01538-19 (PMC6650558; doi:10.1128/mBio.01538-19)
Supplement: TABLE S2 [file mBio.01538-19-st002.pdf]

Table S2: Fetal viability criteria

	Fetal movement/ heartbeat	Fetal size	Skin color
**Viable (live) fetus**	Presence of movement and heartbeat/ visible blood vessels	Normal size for gestational period	Bright pink skin
**Non-viable (dead) fetus**	Absence of movement and heartbeat/ blood vessels not visible	Small size for gestational period and/or significantly smaller than litter-mates and/or fetal reabsorption	Pale opaque skin
