# Supplementary material for: Brucella abortus Infection of Placental Trophoblasts Triggers Endoplasmic Reticulum Stress-Mediated Cell Death and Fetal Loss via Type IV Secretion System-Dependent Activation of CHOP
Source: mBio. 2019 Jul 23;10(4):e01538-19. doi: 10.1128/mBio.01538-19 (PMC6650558; doi:10.1128/mBio.01538-19)
Supplement: TABLE S3 [file mBio.01538-19-st003.pdf]

Table S3: Placental histopathology scoring criteria (reproduced from Ref. 14)

Score	*Granuloma*	*Neutrophils*	*Necrosis*	*Vascular lesions*
0	Absent	Absent	Absent	Absent
1	Mild focal to multi-focal granuloma formation	Mild focal to multi-focal neutrophil infiltration	Mild focal to multi-focal trophoblast death and/or coagulative necrosis	Mild focal to multi-focal hemorrhage and/or thrombi
2	Moderate focal to multi-focal granuloma formation	Moderate multi-focal neutrophil infiltration	Moderate multifocal trophoblast death and/or coagulative necrosis	Moderate multi-focal hemorrhage and/or thrombi
3	Severe focal to multi-focal granuloma formation	Severe multi-focal to diffuse neutrophil infiltration	Severe multifocal to diffuse trophoblast death and/or coagulative necrosis	Severe multi-focal to diffuse hemorrhage and/or thrombi
